# Fabrication of ultrahigh-density nanowires by electrochemical nanolithography

**DOI:** 10.1186/1556-276X-6-444

**Published:** 2011-07-11

**Authors:** Feng Chen, Hongquan Jiang, Arnold M Kiefer, Anna M Clausen, Yuk-Hong Ting, Amy E Wendt, Bingjun Ding, Max G Lagally

**Affiliations:** 1 Xi'an Jiaotong University, Xi'an, Shaanxi 710049, China; 2 University of Wisconsin-Madison, Madison, WI 53706, USA

## Abstract

An approach has been developed to produce silver nanoparticles (AgNPs) rapidly on semiconductor wafers using electrochemical deposition. The closely packed AgNPs have a density of up to 1.4 × 10^11 ^cm^-2 ^with good size uniformity. AgNPs retain their shape and position on the substrate when used as nanomasks for producing ultrahigh-density vertical nanowire arrays with controllable size, making it a one-step nanolithography technique. We demonstrate this method on Si/SiGe multilayer superlattices using electrochemical nanopatterning and plasma etching to obtain high-density Si/SiGe multilayer superlattice nanowires.

## Introduction

Low-dimensional systems are of high interest because their unique properties can improve device performance in a range of applications, including optics [1,2], mechanics [3], microelectronics [4], and magnetics [5]. These systems have enhanced surface and quantum confinement effects caused by the large surface-to-volume ratio and small size, making them dramatically different from their bulk counterparts. Superlattice nanowires have the potential to improve the performance of thermoelectronics [6-9], small sizes have lower thermal conductivity [8,9], and they can be made at a high density, thus providing improved performance.

Generally, there are two major approaches in the fabrication of nanostructures: bottom-up [10] and top-down [11]. Among the various bottom-up methods, vapor-liquid-solid (VLS) growth is one of the most popular and is used to grow nanostructures such as nanowires [12-14]. VLS growth uses a catalytic liquid-alloy phase that can rapidly adsorb a vapor to supersaturation levels, in which crystal growth can subsequently occur from nucleated seeds at the liquid-solid interface. It is a relatively simple method and yields a large quantity of nanowires from a single growth. However, the requirement of metal particle catalysts risks contaminating the nanowires [15], and it is not easy to control the density and nanowire size, shape, and crystal orientation simultaneously [16]. Additionally, twin boundaries normally form in the VLS growth, which may affect the subsequent nanowire performance [17].

Typically, top-down approaches involve lithography, which defines the lateral size and shape of the final structure using an electron/photon-sensitive resist as mask material. Examples are electron beam lithography [18] and X-ray nanolithography [19]. For example, Zhong *et al*. have reported ordered SiGe/Si superlattice pillars combining holographic lithography, molecular beam epitaxy (MBE) growth, and wet chemical etching [20]. Although e-beam and X-ray lithographies create uniformly distributed and ordered templates for further top-down processing, they are expensive and time consuming. They also require several processing steps, involving photoresist deposition/removal and chemical or ion beam etching. Other approaches utilize self-assembling [21] structures such as block copolymers [22] or anodic aluminum oxide as masks [13,23]. The outputs of self-assembling techniques are uniform in size and ordered over a large scale; however, they usually require additional deposition, baking, etching, and stripping processes.

Instead of a patterned photoresist, it is also possible to use nanoparticles (NPs) as a nanolithography mask. NPs can be prepared by electrochemical deposition (ECD), an easy, fast, economical, and straightforward way to deposit materials directly on top of semiconductors [24] or metals [25-27]. To the best of our knowledge, ECD of NPs has not been reported in top-down semiconductor nanostructure fabrication.

We deposit silver nanoparticles (AgNPs) in sizes of tens of nanometers, using pulsed-current driven ECD. By adjusting the deposition conditions, we achieve high-density AgNPs with uniform size and spacing. The resulting one-step electrochemically deposited AgNPs are very robust and can survive further processing. Therefore, they can be used as a hard mask for plasma etching or as a metal-assisted etching mask [23]. By using this mask in combination with chemical vapor deposition (CVD) growth and plasma etching, we are able to fabricate ultrahigh-density (6.2 × 10^10 ^cm^-2^) Si/SiGe superlattice nanowire arrays over a large area, with individual wires < 30 nm in diameter and approximately 200 nm in length.

### Experimental details

**Figure 1:**
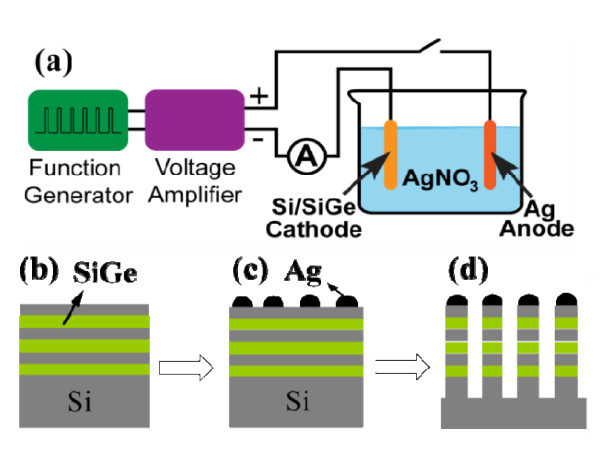
**Schematic diagram of the AgNP-based nanowire fabrication sequence**. **(a) **Experimental setup of ECD and fabrication steps of superlattice nanowires. **(b) **CVD growth of 2D superlattice. **(c) **ECD of NPs. **(d) **Plasma etching to create superlattice nanowires.

The ECD system used in our experiment is schematically shown in Figure 1a. It consists of a function generator (Agilent 33220A, Agilent Technologies, Inc., Santa Clara, CA, USA), a voltage amplifier (Agilent 33502A, Agilent Technologies, Inc., Santa Clara, CA, USA), a glass beaker, Ag foil (99.9%, Aldrich, Sigma-Aldrich, St. Louis, MO, USA) as the anode, and the semiconductor substrate as the cathode.

The Si/SiGe superlattice wafer is prepared using low-pressure CVD. We grow a ten-period Si/Si_0.82_Ge_0.18 _superlattice structure on Si wafers (Si(001), p-type, nominal doping density of 10^15 ^cm^-3^), and cap the superlattice with Si (Figure 1b). The layer thickness is 10.8 nm for Si and 7.0 nm for the SiGe alloy, as confirmed by X-ray diffraction (XRD, PANalytical X'Pert MRD, PANalytical, Inc., Westborough, MA, USA). Both layers are grown at 580°C, with silane and germane as precursors. The root mean square surface roughness measured by atomic force microscopy (AFM Digital Instrument Nanoscope IV, Veeco Instruments, Santa Barbara, CA, USA) after CVD growth of all the layers is 0.7 nm.

Before performing ECD, we dip the as-grown superlattice wafer in hydrofluoric acid to remove the native oxide on the top Si layer. After rinsing in deionized water (DI) for 5 min, we quickly immerse it into the AgNO_3 _solution (1 × 10^-4 ^M).

We use pulsed current as the deposition driving force to deposit nanoparticles because this approach is very controllable when depositing a small amount of material. The pulsed signal consists of a long period (*T*) with a short pulse length (*τ*). Various immersion times (*t*) and pulse lengths (*τ *= 1 ms to 0.5 s) were tried in the experiment in order to obtain AgNPs with uniform small size and high density.

After AgNP deposition, plasma etching is performed to produce superlattice nanowires. The substrate, with the AgNPs acting as a hard mask, is etched by a high-density helicon plasma tool equipped with a diode laser interferometer for *in situ *etch depth measurement (Figure 1d) [28]. A gas mixture of SF_6_/C_2_H_2_F_4 _is used. Source power and bias voltage are finely tuned to obtain a vertical etch profile. Using this system, we are able to etch out nanowires up to several microns in length [29].

## Results and discussion

**Figure 2:**
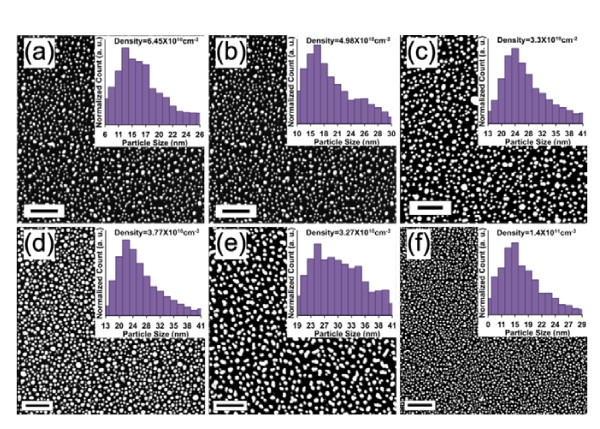
**Scanning electron microscopy images and size distribution histograms**. Scanning electron microscopy images and size distribution histograms of immersion time and pulse length dependent size distribution of AgNPs. From **(a-e) **the pulse length *τ *= 0.5 s with different immersion times: (a) *t *= 10 s, (b) *t *= 20 s, (c) *t *= 30 s, (d) *t *= 40 s, and (e) *t *= 50 s. **(f) **Pulse length *τ *= 1 ms and *t *= 50 s. Insets are histograms of the particle diameter. The scale bars in all Figures are 200 nm.

Figure 2 shows scanning electron microscopy (SEM, Zeiss, LEO-1530, Carl Zeiss SMT GmbH, Oberchoken, Germany) images of AgNPs after ECD under various conditions. In the experiment, we fix the pulse period *T *to be 1 s. The size and density of the AgNPs vary with the immersion time and pulse length. The pulse length *τ *is first fixed to 0.5 s in order to study the result for different immersion time *t*. We vary the immersion time from 10 s to 50 s in steps of 10 s. For *t *= 10 s, the average NP size is around 20 nm in diameter, with 14-17 nm NPs the most prevalent (Figure 2a). The size distribution remains qualitatively the same with increasing immersion time up to 40 s, but the average and most prevalent sizes increase. For *t *= 20 s, the average size is 25.4 nm, and the most prevalent size range is 17-20 nm (Figure 2b). Finally, nanoparticles with diameters of 29 ± 4 nm are achieved at an immersion time of 40 s, as shown in Figure 2d. When the immersion time reaches 50 s, however, the sizes become random and range from 25 to 37 nm (Figure 2e).

For a much shorter pulse length, *τ *= 1 ms, the general trend of size distribution is the same, but particles are much smaller and the uniform size distribution lasts to longer immersion times. For comparison, the results of the 50-s immersion time are shown in Figure 2f.

The applied electric field is the driving force of the Ag^+ ^reduction reaction. Each positive pulse applied on the electrode drives Ag^+ ^ions towards the cathode, here the Si surface. The pulse length of the pulse determines the number of adatoms arriving on the surface. Because the applied voltage (20 V) is much higher than the overpotential (300 mV), the effects of the space charge layer and the Helmholtz layer can be ignored when considering the potential profile across the substrate/solution interface [30].

**Figure 3:**
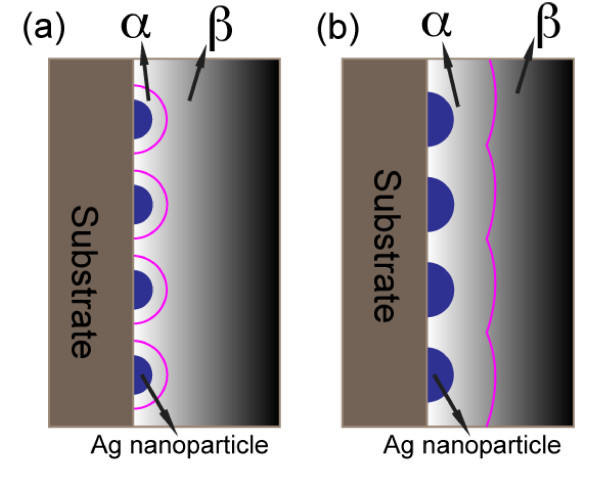
**Different ion concentrations cause interparticle interaction transition**. **(a)** Weak interaction at higher concentration (thinner depletion layer), **(b) **strong interparticle interaction at lower concentration (thicker depletion layer). α is the depletion layer and β is the diffusion layer. The purple lines indicate the interface between diffusion layer and depletion layers.

**Figure 4:**
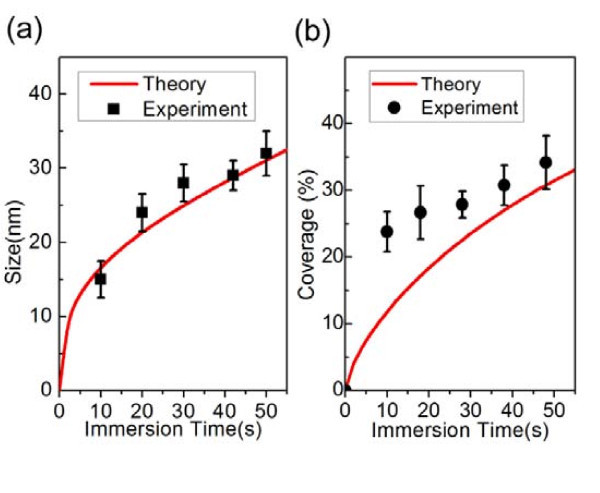
**Ag nanoparticle size and coverage as a function of immersion time**. Ag nanoparticle **(a) **size and **(b) **coverage as a function of immersion time. Symbols are experimental values; solid lines are prediction.

**Figure 5:**
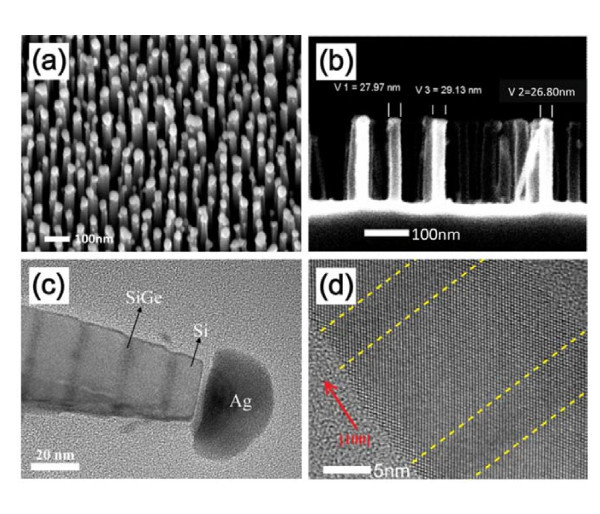
**SEM and HRTEM images of etched nanowires**. **(a) **45°-tilted view of nanowires with Ag nanoparticle caps. **(b) **Side view of nanowires, the Ag particles are removed by HNO_3_. Some nanowires are bent because of air blow-drying of the sample after the dip in HNO_3 _and rinsing in DI water. **(c) **Cross-sectional TEM image of superlattice nanowires with Ag particles retained. The darker lines are the SiGe alloy. The lateral width of the SiGe layer is smaller than the Si layer because Ge etches faster than pure Si in a SF_6_/C_2_H_2_F_4 _plasma. **(d) **HRTEM image showing the atomic detail of different sections. The dashed lines guide the eye to the interfaces between Si and SiGe.

Because of the weak chemical interaction between the adatoms and the substrate, nucleation and growth of Ag on Si occurs via a Volmer-Weber mode [31]. For a pulse with long pulse length (such as 0.5 s), the nucleation is an instantaneous process [31], and the growth is diffusion-limited [32]. In the first pulse, the nucleation density reaches its maximum during the first few milliseconds and passes the peak value thereafter, thus nucleus coarsening follows the nucleation in order to reduce the total free energy of the system. At the moment nucleation is completed, the ion-depleted layers (the solution layer near the surface where there are no ions; it is thinner than the diffusion layer) surrounding each nucleus are well separated from each other (Figure 3a). As a result, the growth of a nucleus is not influenced by the growth of neighboring particles. Therefore, in this "weak interparticle interaction" limit, particles grow at equal rates. However, reduction of ions to atoms causes the global ion concentration in the diffusion layer to decrease. The 0.5-s pulse-off time will allow the depleted layer to be repopulated by ions diffusing from far-off electrode regions. This reasoning explains why the particle density decreases with increasing immersion time (*t *≤ 40 s) while still remaining quasi-uniform. The particle density decreases from 6.28 × 10^10 ^cm^-2 ^in Figure 2a to 5 × 10^10 ^cm^-2 ^in Figure 2b and stabilizes around 3.5 × 10^10 ^cm^-2 ^in Figure 2c, d. The density reduction rate is slowed at longer immersion time because the solution ion concentration reduction increases the diffusion layer thickness.

Low ion concentration or long immersion time can cause a transition from weak interparticle interaction to strong interaction. At long immersion times (*t *> 40 s in our experiment), depletion layers at adjacent particles merge to create an approximately planar diffusion layer across the entire surface (Figure 3b); this strong interparticle interaction makes the flux of ions per unit area on the surface more uniform. Because the nucleation density is locally variable, densely nucleated regions are therefore expected to have a slower growth rate than regions of the same size but encompassing a smaller number of nanoparticles. Thus, when the immersion time reaches 50 s the size distribution becomes less uniform (Figure 2e). At the same time, Ostwald ripening decreases particle size uniformity as smaller islands are eliminated by the larger ones [33].

By reducing the pulse length, we achieve higher particle density and smaller particle size while still maintaining good size uniformity. In Figure 2f, the AgNPs have a density of 1.4 × 10^11 ^cm^-2^, which is almost twice the density for *τ *= 0.5 s (Figure 2e), while the relative standard deviation of size is only 42%.

The AgNPs are crown-shaped rather than hemispherical. If we assume that the deposited atoms are (almost) all forming nanoparticles and the distribution of sizes do not vary from what is observed in the SEM image, the total number *N *of deposited nanoparticles at equilibrium is given by [30].(1)

where *r *is the radius of the NP, *N*_A _is Avogadro's constant, *M*_0 _is the molar mass of Ag, *g *is the bulk Ag density, *I *is the measured current, and *q*_0 _is the electron charge. From Equation 1, the diameter of AgNPs can be expressed as a function of immersion time and pulse length:(2)

If we assume the AgNPs are hemispherical, the percentage of the Si surface covered by AgNPs is:(3)

where *n/s*_0 _is the particle density on the SEM image area.

Inserting the experimental pulse length *τ *= 0.5 s into Equation 2 and Equation 3, we can plot the size of the particles and the coverage of the substrate as functions of the immersion time, as shown in Figure 4a,b, respectively. The most common particle size agrees with theory very well (Figure 4a). However, the average particle size is larger than the most common particle size, indicating variation in the shape of the AgNPs in the ECD. When the immersion time is short, the particles are crowns instead of hemispherical. As the immersion time increases, the height of the particle increases, and this shape change results in the experimental coverage getting closer to the theory curve, in which we assume the particles to be perfect hemispheres, as shown in Figure 4b. The NPs will finally turn into a continuous film if a long enough immersion time is used [30].

Using the AgNPs as a mask, vertically aligned superlattice nanowires are etched, as shown in Figure 5, which shows both scanning electron microscopy and transmission electron microscopy images. If a single etching time is too long, because sidewall etching occurs simultaneously, the top of the nanowires (closest to the plasma source) will be etched away and the nanowire will form a tapered structure (Figure 5c). This problem can be overcome by depositing a fluorocarbon film (using C_4_F_8 _as precursor) on the sidewall during etching [34]. It protects the sidewall from being further etched, and it can be removed with an O_2 _plasma or HF afterward. The details of deep reactive-ion etching (RIE) can be found in Ref. [35].

Using high-resolution transmission electron microscopy (HRTEM, Philips, CM200UT, Philips Electron Optics BV, Eindhoven, The Netherlands), we can explore the layered structure of the nanowires. Figure 5c, a relatively low-magnification image, shows the periodic variation in brightness representative of the layers. The darker regions are the SiGe alloy because Ge scatters electrons more strongly. We can clearly see the interfaces between Si and SiGe layers. Figure 5d demonstrates that the nanowires are single crystals, as we expect from the MBE growth. In the VLS growth of nanowires, normally twin boundaries are observed [36]. With our method, this problem is eliminated because the starting material is a CVD-grown single-crystalline 2D superlattice.

The SiGe alloy etches faster than pure Si in a SF_6_/C_2_H_2_F_4 _plasma, giving the edge of the wire a scalloped appearance, effectively introducing surface roughness to the sidewall. The etching process does not affect the crystallographic properties of the superlattice. The surface roughness may result in enhanced phonon scattering [37].

Si and Ge nanowires have been considered as potentially good thermoelectric materials, because of the reduced thermal conductivity at small dimensions. Superlattice nanowires have even greater potential because of the band offset between Si and SiGe [37]. Thus, electric conductivity is possibly improved through the superlattice structure [38,39]. The combination of excellent superlattice with edge roughness of our etched nanowires may therefore bring higher thermoelectric efficiency for group IV materials than has been possible so far.

## Conclusion

In this paper, we introduce a one-step nanolithography method to fabricate quantum wires with diameters down to 15 nm using electrochemically deposited AgNPs. The AgNP density obtained is as high as 1.4 × 10^11 ^cm^-2 ^with coverage up to 37% over a large area. By adjusting the pulse length and immersion time, the size and density of AgNPs can be well controlled. We demonstrate that these high-density AgNPs can be used as a hard etching mask to fabricate vertically aligned Si/SiGe superlattice nanowires. Because the method does not need lithography to define the pattern, it is much less expensive and can make very small patterns that may have considerable use even if the pattern is not totally uniform. The size and coverage of etched nanostructures only depend on the AgNPs. The method can be used with substrates of any material as long as it conducts sufficiently to form a cathode for the electrochemical process, and a proper etch chemistry to which Ag is resistant is available. The AgNP mask can be used in both metal-assisted etching to etch Si and SiGe and RIE to etch most other semiconductor materials. The method has the potential to make nanowires of different materials, as well as different orientations. It should be very useful in making devices requiring nanowires, such as nanothermoelectronic devices, that require a small size, narrow dispersion, and high density.

## Competing interests

The authors declare that they have no competing interests.

## Authors' contributions

FC and HJ designed and carried out the study and performed all the ECD. AMK provided expertise on and participated in the CVD growth. AMC provided expertise on and assisted with the TEM characterization. Y-HT and AEW provided expertise on and participated in the plasma etching. FC and MGL prepared the MS. All authors read and approved the final manuscript.
